# Complete genome of a novel mycobacteriophage WXIN isolated in Wuhan, China

**DOI:** 10.1186/s12863-024-01244-8

**Published:** 2024-06-18

**Authors:** Haoming Wu, Wenxin Li, Chi Zeng, Jiaxin Li, Huan Wu

**Affiliations:** 1 Pilot Base of Food Microbial Resources Utilization of Hubei Province, School of Life Science and Technology, Wuhan Polytechnic University, Wuhan, 430023 China; 2 Department of Laboratory Medicine, Wuhan Children’s Hospital, Tongji Medical College, Huazhong University of Science and Technology, Wuhan, 430019 China

**Keywords:** Mycobacteriophage, *Mycobacterium septicum*, Complete genome

## Abstract

**Objectives:**

The rising of antibiotic resistance has sparked a renewed interest in mycobacteriophage as alternative therapeutic strategies against mycobacterial infections. So far, the vast majority of mycobacteriophages have been isolated using the model species *Mycobacterium smegmatis*, implying an overwhelming majority of mycobacteriophages in the environment remain uncultured, unclassified, and their specific hosts and infection strategies are still unknown. This study was undertaken to isolate and characterize novel mycobacteriophages targeting *Mycobacterium septicum*.

**Data description:**

Here a novel mycobacteriophage WXIN against *M. septicum* was isolated from soil samples in Wuhan, China. Whole genome analysis indicates that the phage genome consists of 115,158 bp with a GC content of 61.9%. Of the 260 putative open reading frames, 46 may be associated with phage packaging, structure, lysis, lysogeny, genome modification/replication, and other functional roles. The limited genome-wide similarity, along with phylogenetic trees constructed based on viral proteome and orthologous genes show that phage WXIN represents a novel cluster distantly related to cluster J mycobacteriophages (genus *Omegavirus*). Overall, these results provide novel insights into the genomic properties of mycobacteriophages, highlighting the great genetic diversity of mycobacteriophages in relation to their hosts.

## Objective

The emergence and rapid spread of drug resistance, especially multidrug-resistant (MDR) and extensively drug-resistant (XDR), poses a great challenge to the clinical control for pathogenic mycobacteria, particularly *Mycobacterium tuberculosis (M. tuberculosis)* infection [1]. In recent years, phage treatment has undergone a revitalization as a promising strategy against antimicrobial resistance. Phages, formerly bacteriophages, are natural enemies of bacteria and are believed to be the most abundant organisms on the planet [2]. Unlike antibiotics, phages are characterized by self-replication, high host specificity, biofilm degradation, and low toxicity to humans [3, 4]. Several successful clinical trials have been reported to treat patients with disseminated drug-resistant *M. chelonae* and *M. abscessus* infections using naturally occurring phages and/or their genetically engineered derivatives [3, 5, 6].

Since the first mycobacteriophage was identified in 1947 from soil [7], more than 12,000 mycobacteriophages have been isolated from different sources, of which approximately 2,000 have their complete genomes sequenced (https://phagesdb.org). These mycobacteriophages are categorized into 30 different types of clusters and at least 10 singletons. Due to the non-pathogenicity, rapid growth, and similarities with other mycobacteria, *M. smegmatis* has been used as a suitable model for studying the pathogenesis of mycobacteria and for initial screening for mycobacteriophages [8]. Hence, almost all known mycobacteriophages were isolated using the *M. smegmatis* mc^2^155 as host. Further isolation and characterization of mycobacteriophages against different mycobacterial species in different environments will not only enrich our knowledge of phage genetics, ecology, and evolution, but also provide rich phage resources for genetic engineering. *M. septicum* is a rapidly growing non-tuberculosis mycobacteria associated with *Mycobacterium fortuitum* group (MFG) [9]. In this study, a novel mycobacteriophage WXIN against *M. septicum* was isolated from soil samples in Wuhan, China. The results provide some new insights into genomic characteristics of mycobacteriophage.

## Data description

The mycobacteriophage WXIN was originally isolated from soils collected in Wuhan, China, using *M. septicum* as host. Phage genomic DNA was extracted using the λ Phage genomic DNA extraction kit (Beijing Baiaolaibo Biotechnology Co., Ltd, Beijing, China). Sequencing libraries were prepared using the NEBNext Ultra DNA library prep kit (New England Biolabs, USA) and sequencing was performed on the Illumina NovaSeq 6000 platform to generate 4,191,323 paired-end reads (2 × 150 bp). The quality of the sequencing reads was evaluated using FastQC v0.12.1 (https://www.bioinformatics.babraham.ac.uk/projects/fastqc/) and was assembled de novo using Megahit v1.2.9 [10]. Genome termini were identified by PCR using primers (WT: 5’-CACATGTCGGCGTGACGT-3’ and WW: 5’-CGTCATTAATGTCCCCCTCG-3’) facing off the contig ends to produce an approximately 400 bp product, and the PCR product was submitted for Sanger sequencing by Sangon Biotech (Shanghai, China). In total, the complete genome of mycobacteriophage WXIN was 115,158 bp with a GC content of 61.9%. Genome was annotated using the RAST server (https://rast.nmpdr.org/). The predicted proteins were assigned putative functions by HHpred [11] and BLASTp-searching against the NCBI non-redundant (nr) database (https://www.ncbi.nlm.nih.gov). A total of 260 putative open reading frames (ORFs) were identified in the genome, with 184 on the sense strand and 76 on the antisense (Data file 1) [12]. Among them, 46 ORFs could be functionally annotated and classified into modules involving in the packaging, structure, virion release, lysogeny, genome modification and replication (Data file 2) [12]. Two tRNA genes were identified by tRNAscan SE 1.21 (http://lowelab.ucsc.edu/tRNAscan-SE/). No virulence or drug resistance-related gene was found using the Virulence Factor Database (VFDB, http://www.mgc.ac.cn/VFs/) and the Comprehensive Antibiotic Resistance Database (CARD, https://card.mcmaster.ca/).

Proteomic trees generated by ViPTree [13] indicates that WXIN is mostly related to cluster J mycobacteriophages (genus *Omegavirus*) and likely represents a novel mycobacteriophage cluster (Data file 3) [12]. Genome-wide comparisons by VIRIDIC (https://rhea.icbm.uni-oldenburg.de/viridic/) reveals extremely low sequence similarity between WXIN and cluster J mycobacteriophages (Data file 4, 21.2–24.0% nt identity) [12]. In addition, 15 single-copy orthologous genes are shared by WXIN and the related cluster J, X and E mycobacteriophages, as indicated by Orthofinder v2.2.7 through all-to-all BLASTp analysis [14], including the terminase large subunit (ORF7), tape measure protein (ORF29), minor tail proteins (ORF31 and ORF33) and lysine protein A & B (ORF43 and ORF45) and several hypothetical proteins (ORF53, ORF82, ORF128, ORF130, ORF146, ORF175, ORF184, ORF193 and ORF194). The average amino acid identity (AAI) of the 15 orthologous genes range from 40.7 to 64.1% between WXIN and other related mycobacteriophages (Data file 5) [12]. Phylogenetic analysis based on the concatenated protein sequences of the 15 orthologous genes also revealed similar tree topology with that of ViPTree analysis using IQ-TREE v1.6.5 (Data file 6) [12, 15].

**Table 1:** Overview of data files/data sets

Label	Name of data file/data set	File types (file extension)	Data repository and identifier (DOI or accession number)
Data file 1	Circular genome annotation of mycobacteriophage WXIN	Portable Document Format file (.pdf)	Science data bank (10.57760/sciencedb.16539) [12]
Data file 2	Predicted genes of mycobacteriophage WXIN	MS Excel file (.xlsx)	Science data bank (10.57760/sciencedb.16539) [12]
Data file 3	Proteomic tree by ViPTree	Portable Document Format file (.pdf)	Science data bank (10.57760/sciencedb.16539) [12]
Data file 4	Intergenomic similarities between WXIN and 10 Cluster J mycobacteriophages	Portable Document Format file (.pdf)	Science data bank (10.57760/sciencedb.16539) [12]
Data file 5	AAI values of 15 orthologous genes	Portable Document Format file (.pdf)	Science data bank (10.57760/sciencedb.16539) [12]
Data file 6	ML phylogenetic Inference of 15 orthologous genes	Portable Document Format file (.pdf)	Science data bank (10.57760/sciencedb.16539) [12]
Data set 1	Mycobacteriophage WXIN, complete genome	Genbank file (.gbk)	NCBI Nucleotide, https://identifiers.org/nucleotide:OR813930 [16]
Data set 2	Raw sequencing reads of Mycobacteriophage WXIN	SRA file (.sra)	Sequence Read Archive (https://identifiers.org/ncbi/insdc.sra:SRP492847) [17]

## Limitations

This data note was limited to the description of mycobacteriophage WXIN. A larger collection is needed to help us better understand the genetic characteristics of mycobacteriophages for *Mycobacterium septicum*.

## Data Availability

The genomic sequence described in this Data note can be freely and openly accessed on NCBI GenBank under accession number OR813930. Raw sequence reads are available from NCBI under BioProject PRJNA1082475.
